# Materials for Separating Teeth

**Published:** 1884-12

**Authors:** D. Genese


THE
AMERICAN JOURNAL
OF
DENTAL SCIENCE.

VOL. XVIII. THIRD SERIES—DECEMBER, 1884. No. 8.

ARTICLE I.

### MATERIALS FOR SEPARATING TEETH.

[Read before the Maryland State Dental Society.]

BY D. GENESE, D. D. S.

The continued failure of most appliances for separating teeth previously to filling or stopping, is caused by the following circumstances:

The teeth being narrower at the neck than at their cutting edges, any material at present used for the purpose of separating them has the tendency of drawing or working its way towards the margin of the gum and producing so much irritation that to some delicate and nervous persons it is impossible to bear the pain long enough to separate the teeth sufficiently to operate upon, thereby causing delay, broken appointments and often a failure in placing in the approximate cavities a perfect filling.

The materials used at present are not under sufficient control to enable the patient to place the wedge in position; those made of wood having to be driven in by force, and giving great pain. Often also splinters entering the tissues of the gum and causing severe inflammation.

Uncooked, or pure rubber, another material used for separating, is not to be relied upon, as the temperature of the mouth will cause it to alter its shape and being very plastic will be forced by the action of mastication between the gum and tooth, perhaps on one side or both and cause a bad ulcerated gum margin, which may bleed upon the slightest touch and spoil a long operation. No definite shape can be given to this material, as the act of cutting it with scissors the only means of shaping it will defeat the object by squeezing it at the wrong place and making not a wedge, but a ball of pulp.

Another material frequently used is cotton wool. This has very little expanding properties, and has to be packed little by little until a sufficient wedge is formed to make a compress, but should this material remain over thirty hours in the mouth, and less in some, it will decompose and cause extensive mischief.

Wedges are also made by cutting pieces from different sized rubber gas tubing.

This system cannot too soon be condemned, as the price it is sold at should convince us that it is not a material pure enough to place in the mouth of any person. The same objection also exists as to cutting pieces from uncooked rubber, viz: The imposibility of shaping it as desired, even if time and care is expended upon the effort, while the pigment in it destroys very quickly any elasticity it may posess.

The above-mentioned materials have so frequently been inadequate to accomplish the desired result that a double wedge of steel acting both from the palatine and lubial surface of the tooth by a set screw has been resorted to, but it is a cruel and painful operation, and in the opinion of many eminent men is considered to cause permanent injury to the part acted upon.

After a series of experiments I have found a preparation, and the means of adapting it, so that the dentist can accomplish the desired object of separating the teeth to exactly the distance required for the operation with little inconvenience to the patient and comparative freedom from pain

It is made of pure rubber only and *vulcanized in steam* (not acid cured) and without *sulphur*. Steel moulds are used under great pressure thereby giving great density to the rubber and prevent it altering its shape, but the most important point is to have the strips made *triangular* varying in thickness and depth, and with the edge intended to be near the margin of the gums *smooth* and *round*, unless it is desired to force the margin above a cavity when the *smooth flat* surface may be presented.

It will readily be seen that a wedge so prepared is of great utility.

1. It will not become softened or alter its shape by the fluids of the mouth and is perfectly free from deleterious ingredients.

2. It will always return to its original form and exert a continuous pressure until it has done so, and presenting the greatest surface against the cutting edge of the tooth the gum margin will escape under pressure.

3. It will not be driven too close to the margin in the greatest number of cases (there are always exceptions to every rule), but should such a circumstance occur, the smooth surface of the prepared rubber strips will not irritate nor wound the gum, like rubber cut unevenly, wool, or cotton, which is generally left with ragged edges.

4. The strips of rubber being prepared in consecutive thickness, the teeth can be gradually and almost painlessly separated by the patients themselves, thereby saving many visits to the dentist office and yet being sure of the separation being accomplished.

In applying the wedge to the teeth it is only necessary to stretch the strip of rubber thin enough to pass between the teeth, round edge upward leaving the flat lever with the cutting edge, cutting the surplus so that ⅛ inch extends to both sides of the tooth (do not force it near the gum), it will then by its own force of regaining its original form, divide the teeth and work upward until pressure is no longer exerted.

5. It will not not expand more than its original form in moisture, thereby enabling the dentist to decide the size pieces of rubber to use in rotation, avoiding unnecessary division and displacement of the teeth.

6. Should it be required to force the gum beyond the neck of the teeth, one of which may have an approximal cavity above the margin of the gum it is only necessary to present the broad flat surface of the strip upward and the edges will lay close to the teeth and lift the gum above the cavity without bruising it, and make what has been a difficult operation to accomplish one quite easy, time-saving and free from pain.

